# Clinical educational scholarship: polarized or integrated?

**DOI:** 10.1007/s40037-020-00597-x

**Published:** 2020-06-03

**Authors:** Sophie Park, Anita Berlin, Martina Kelly

**Affiliations:** 1 Research Dept. of Primary Care and Population Health, University College London, London, UK; 2 Institute of Health Science Education, Queen Mary University of London, London, UK; 3 Cumming School of Medicine, University of Calgary, Calgary, Canada

Healthcare education spans a range of institutional ‘homes’, from vocational colleges, practice-based organizations, to the university-based academy. These vary across disciplines, geographical areas, and undergraduate/postgraduate worlds. These ‘homes’ produce a range of different organizational and cultural spaces for scholarship, and related opportunities for promotion and professional recognition. Each legitimize (and de-legitimize) particular activities and achievements as ‘valuable work’, contributing to hierarchical relations both within and across disciplines [1, 2]. We draw upon experience from three professional narratives: each working across undergraduate and postgraduate clinical educational roles in the UK and Canada. We reflect upon these situated journeys in relation to some of the key analytical arguments in Etmanski’s article on ‘Sense-making narratives of scientists working in health professions education scholarship units’ [3].

Many healthcare educational roles require expertise and collaboration *across* disciplinary boundaries. Clinical academics, for example, need to function within fast-changing fields of clinical practice, teaching and research. Scientist academics, similarly, require growth within particular scientific disciplines (e.g. sociology, psychology), while also developing expertise in clinical education. These dual or triple professional dimensions produce a number of challenges and competing priorities, such as keeping up-to-date and maintaining professional accreditation, alongside balancing administrative, teaching and research commitments. As Etmanski et al. highlight [3], these sometimes result in strategic game-playing to re-attribute activities (e.g. supervision and collaborative meetings) as teaching or research, to fulfill institutional requirements across domains. There are, however, important opportunities of integration (e.g. between research and teaching), cross-disciplinary and cross-methodological working, for both individuals and disciplinary fields.

‘*Praxis*’ or knowledge of doing, and ‘scientific’ knowledge are closely enmeshed in both the practice of medicine and clinical education. There have, however, been historical shifts in the organizational positioning and recognition of practical and scientific knowledge as more or less polarized and/or integrated. These support (or not) spaces for the critical implementation of scientific knowledge (about topic and process) within both teaching and clinical practice. Flexner in the US [4], for example, promoted the ‘scientification’ of medical education as distinct from practical knowing [5]. The Dearing Report in the UK [6] in contrast, promoted the ‘scholarship of education’ advocating inclusion of educational activities as markers of academic excellence and quality.

Key technologies (e.g. policies) shape the relationship between teaching and research and their relative polar or integrated positions. Efforts, such as the UK Research Assessment Exercise or the more recent Research Excellence Framework have, for example, contributed to prioritization of research-based activity in many university-based institutions. The UK Teaching Excellence Framework later aimed to counter-balance this hierarchical attention to research. An appetite for institutional recognition of teaching has, however, led to a productive integration of research and teaching in medical education, with burgeoning fields of ‘educational research’ and a reciprocal appetite for implementation of scholarly knowledge within educational practice. Many institutions have responded to and supported these developments in positive ways providing, for example, both teaching and research scholarship promotion pathways, and supporting scholarly professionalization of teachers such as the Higher Education Academy fellowship framework, or postgraduate masters and doctoral programs.

Across clinical and scientist academic careers in healthcare education, the challenge (for both individuals and institutions) remains how to balance and attend to these ever evolving complementary and competing aspects of ‘work’. Most roles include a mix of both the mundane and bureaucratic (meetings, form-filling), with intellectual stimulation (intellectual challenge of consulting with patients; engaging with and producing critical debate in teaching and research; ensuring integration of principles including sustainability, inclusivity and accountability). The challenge of negotiating space between the mundane and stimulating, and promoting personal fulfillment in work, has generated whole genres of professional development, well-being and leadership fields. These are complex and varied, and cross a wide spectrum of individualistic and organizational focus in their efforts to improve quality, well-being, productivity, or scholarly performance in some form. There is still much work to be done in supporting the balancing of multiple hierarchies across clinical education, but it can and should be an exciting field to explore if and when both individual and organization find a mutually productive space in which to thrive.
